# Family caregivers’ experiences in dementia villages: a model for sustainable and meaningful care

**DOI:** 10.3389/frdem.2026.1949032

**Published:** 2026-09-11

**Authors:** Ann Kristin Bjørnnes, Ellen-Karine Grov, Eva Elisabeth Hessevaagbakke, Katrin Lindeflaten, Christine Tschudi-Madsen, Malin Holmström-Rising, Christine Tørris, Johannes Hovland, Daniela Lillekroken

**Affiliations:** 1 Department of Nursing and Health Sciences, University of Southeastern Norway, Borre, Norway; 2 Oslo Metropolitan University, Oslo, Norway; 3 Mid Sweden University, Sundsvall, Sweden

**Keywords:** dementia villages, family caregivers, mixed methods, nursing homes, resident thriving, sustainable dementia care, thriving, wellbeing

## Abstract

**Introduction:**

This study examined family caregivers’ experiences of dementia village care and explored their associations with caregiver reactions and residents’ thriving.

**Methods:**

The study utilized an explanatory sequential mixed-methods design. Thirty family caregivers completed questionnaires assessing caregiver reactions, health-related quality of life, and resident thriving. Subsequently, 16 of these caregivers participated in semi-structured interviews. Quantitative data were analysed using descriptive statistics and correlation analyses, while qualitative data were analysed using thematic analysis.

**Results:**

Family caregivers reported high levels of caregiver esteem and low levels of burden, and residents were perceived as thriving, particularly through positive relationships with staff and high-quality care. Although admission was emotionally challenging, caregivers experienced relief, reassurance, and increased trust. Resident wellbeing was linked to meaningful activities, social engagement, supportive staff, and outdoor access. However, caregivers also reported challenges regarding communication, involvement in care decisions, and balancing resident autonomy with safety. Overall, caregiver experiences reflected an adaptive process marked by both trust and uncertainty.

**Discussion:**

The findings indicate that dementia villages promote resident thriving through supportive environments, social relationships, and meaningful engagement. Family caregivers viewed the model positively and maintained active involvement post-admission, suggesting that dementia villages foster more sustainable care by bridging professional support with ongoing family partnership.

## Introduction

Family members of persons living with dementia constitute an often underrecognized yet indispensable component of person-centred care within dementia care environments (Bastholm-Rahmner et al., 2025). Their emotional engagement, experiential knowledge, and advocacy play a critical role in preserving residents’ identity, dignity, and continuity of life. In these care environments, relatives are active partners who contribute to the social and relational pattern of everyday life (Peoples et al., 2020). In this study, “family caregivers” are defined as unpaid relatives (e.g., spouses, partners, adult children, or other family members) who provide ongoing emotional, practical, and/or decision-making support to a person living with dementia because of their pre-existing family relationship (Happ et al., 2024).

Family caregivers of persons living with dementia experience a substantial and increasing burden, both at the personal and societal levels. Evidence from a large European microsimulation study indicates that the overall burden of informal caregiving is projected to rise by nearly 50% between 2000 and 2050, driven largely by population ageing and increasing prevalence of chronic and neurodegenerative conditions such as dementia (Cattaneo et al., 2025). Dementia-related conditions, including Alzheimer’s disease, are among the fastest-growing contributors to caregiving demands, with projections indicating more than a doubling of associated care burden over time (Cattaneo et al., 2025; Major et al., 2026).

The increasing prevalence of dementia represents a major global public health challenge (World Health Organization, 2026), and has led to the development of innovative care models aiming to enhance autonomy, dignity, and quality of life for people living with dementia (Krier et al., 2023). In Norway, more than 115,000 individuals are living with dementia, and approximately 80% of those requiring long-term care reside in nursing homes (Norwegian Institute of Public Health, 2026). These demographic and care-related challenges have prompted national policy initiatives, including the Dementia Plan 2025, which emphasises dementia-friendly communities and innovative models of care (Norwegian Ministry of Health and Care Services, 2021).

Dementia villages (DVs), also referred to as ‘garden cities’, are inspired by the Dutch Hogeweyk model and represent a shift toward small-scale, person-centred environments (Aldring og helse, 2015). These villages provide opportunities for meaningful activities, social interaction, and access to outdoor spaces, while maintaining safety through environmental design and care technologies (Krier et al., 2023; Roberts, 2023). Because family caregivers often remain actively involved after institutionalization (Ashbourne et al., 2021), their experiences can offer important insights into trust, involvement, and perceptions of care quality in these contemporary care settings (Richardson et al., 2019).

Family caregivers navigate complex roles influenced by factors such as sex, age, education, and cultural background. An intersectional perspective highlights how these factors shape experiences and inequalities (Hengelaar et al., 2023). Furthermore, the concept of thriving, encompassing wellbeing, relationships, activities, and environment (Baxter et al., 2019), is central in evaluating care in DVs. Thriving is a multidimensional construct encompassing residents’ wellbeing, emotional state, engagement in meaningful activities, social relationships, and the quality of the physical and care environment (Bundick et al., 2010). Rather than focusing solely on clinical outcomes, thriving captures how persons experience everyday life within institutional care (Bergland et al., 2014).

Implementing DVs as a dementia care model involves significant transitions, not only for residents but also for their family caregivers. According to Meleis’ Transition Theory, transitions are complex processes that occur when persons experience changes in health, roles, relationships, or environments, often accompanied by uncertainty and vulnerability (Meleis, 2010). For family caregivers, this transition often entails a shift from being the primary provider of care to assuming a new, and sometimes less clearly defined role (Richardson et al., 2019). This process may entail emotional strain, renegotiation of responsibilities, and changes in involvement in everyday care (Rosenberg et al., 2026).

Although admission to long-term care may ease some of the practical demands of caregiving, the burden often persists in new forms. Family caregivers continue to experience emotional responsibility, concerns about their relatives’ well-being, and challenges adapting to their role in a new care context (Ashbourne et al., 2021; Gonella et al., 2026). Family caregivers of people with dementia are at increased risk of experiencing loneliness, stress, reduced quality of life, and negative health outcomes (Victor et al., 2021; Monachou et al., 2025).

Within the context of DVs, sustainability extends beyond economic viability to encompass social, relational, and care-related dimensions. A sustainable model of dementia care supports the long-term well-being of both residents and family caregivers while maintaining high-quality, person-centred care (Krier et al., 2023). This involves creating opportunities for autonomy, meaningful daily activities, social participation, and a sense of belonging for people living with dementia (Tierney et al., 2023). Sustainability also requires recognizing and supporting the contributions of family caregivers, whose involvement strengthens continuity and individualized care. Given the growing reliance on informal caregiving, sustainable dementia care depends on balancing formal services with relational resources in ways that are feasible, equitable, and ethically sound (Park et al., 2025). In this regard, DVs represent a potentially sustainable care model by integrating environmental design, social engagement, and family involvement to promote quality of life over time (Park et al., 2025).

While existing research has primarily focused on organisational aspects, architectural design, and healthcare personnel’s experiences in DVs (Frimpong et al., 2024; Lillekroken et al., 2026), less attention has been paid to the perspectives of family caregivers. Understanding how family caregivers experience this care model is essential for evaluating its quality and sustainability. The aim of this study is to explore family caregivers’ experiences of having a relative living in a DV, with particular attention to: (i) how family caregivers navigate the transition into DV care, (ii) how family caregiver burden is experienced and transformed following institutionalisation, and (iii) how family caregivers perceive residents’ wellbeing and thriving within the DV context.

## Methods and materials

### Design

This study employed an explanatory sequential mixed methods design (Creswell and Plano Clark, 2023) and was reported in accordance with the Good Reporting of A Mixed Methods Study (GRAMMS) guideline (O’Cathain et al., 2008). The study was conducted in two Norwegian DVs encompassing 242 dementia care places distributed across small-scale living units, each accommodating primarily 6–8 residents.

### Sample and recruitment

The caregiver coordinator from two Norwegian DVs invited family caregivers of residents to attend information meetings at each site. During these meetings, the researchers presented the study aim, data collection procedures, and participation requirements. The researchers also displayed study information on noticeboards within the DVs. After the meetings, researchers emailed family caregivers written study information and a link to the survey. Those interested in participating in an individual interview were invited to indicate their interest through the survey. A purposive sampling strategy was used to recruit family caregivers with firsthand experience of the DV care model.

### Data collection

Data were collected between November 2024 and May 2025. Quantitative data were collected through an online survey administered via Nettskjema, a secure web-based platform for collecting and storing research data. The survey included validated instruments and demographic questions.

The thriving of older people assessment scale (TOPAS) is a validated 32-item instrument that assesses perceived thriving in long-term care settings and can be completed by family caregivers as proxy respondents (Bergland et al., 2014). Items are rated on a 6-point Likert scale and summed to generate total and factor scores ranging from 32 (lowest possible level of thriving) to 192 (highest possible level of thriving). Previous studies have demonstrated good agreement between proxy and self-ratings and strong internal consistency (Bergland et al., 2014). The instrument has been widely used to assess resident thriving and to examine factors associated with thriving in long-term care environments (Bergland et al., 2015; Björk et al., 2017; Patomella et al., 2016; Santana-Berlanga et al., 2024).

The Caregiver Reaction Assessment (CRA) is a validated 24-item instrument that measures both positive and negative aspects of caregiving across five domains: self-esteem, lack of family support, financial impact, impact on daily schedule, and impact on health (Given et al., 1992). Items are rated on a 5-point Likert scale, with higher scores indicating greater caregiver burden, except for the self-esteem domain where lower scores indicate higher burden. The CRA has demonstrated acceptable reliability in Norwegian caregiver populations, including family caregivers of people with dementia (Grov et al., 2006; Mogensen et al., 2008).

The EQ-5D is a standardized and widely used measure of health-related quality of life, comprising five dimensions (mobility, self-care, usual activities, pain/discomfort, and anxiety/depression) and a visual analogue scale (EQ-VAS) for self-rated health (Garratt et al., 2022). It provides both a descriptive health profile and a single index value.

A demographic questionnaire was used to collect background information about family caregivers and residents. Family caregiver variables included age, sex, education, employment status, income, relationship to the resident, place of residence, and frequency of visits. Information about residents included age, sex, duration of dementia diagnosis, and length of stay in the DV.

The survey also included an invitation to participate in a follow-up interview. Interested family caregivers were asked to provide their contact details. Subsequently, the research team contacted interested family caregivers by email and invited them to participate in an interview at a location of their choice. Interviews were conducted in a variety of settings, including the DV, participants’ homes, cafés, online, and at the university where the researchers were based. Interviews lasted 30–90 min, averaging 50 min.

The sample size was considered sufficient based on the concept of information power, which suggests that the more relevant information participants hold regarding the study aim, the fewer participants are required (Malterud et al., 2016). Given the specific study aim, the relatively homogeneous sample of family caregivers with direct experience of DV care, and the rich, in-depth interview data, the sample was judged sufficient to address the research questions.

The qualitative data were collected through semi-structured interviews. All interviews were audio-recorded using the Diktafon smartphone application. The application securely encrypted the recordings and transferred them to Nettskjema, where they were stored and automatically transcribed. Each interview was conducted by two researchers, with each pair conducting at least two interviews. The interviews were guided by a semi-structured interview guide developed by the researchers, drawing on their expertise and prior research on family caregiver involvement and experiences in dementia and nursing home care (Tasseron-Dries et al., 2023; Gonella et al., 2022). Interviews explored family caregivers’ experiences of caregiving, safety, collaboration with staff, technology use, thriving, and caregiver burden.

### Analyses

Descriptive statistics were used to summarize participant characteristics, resident characteristics, caregiver reactions (CRA), and thriving outcomes (TOPAS-Proxy). Continuous variables are presented as means and standard deviations (SD), while categorical variables are presented as frequencies and percentages. Domain scores for the CRA and TOPAS-Proxy were calculated as mean scores across the items included in each domain. Spearman’s rank-order correlations were used to examine associations between overall thriving and the TOPAS-Proxy and CRA domain scores. We also explored correlations among CRA domains. Spearman correlations were chosen because of the small sample size and ordinal-level measurements. Statistical significance was set at *p* < 0.05.

Qualitative data were analysed using reflexive thematic analysis following the six-step approach described by Braun and Clarke (2006, 2022): familiarization with the data through repeated reading of the transcripts, generation of initial codes, development of themes by identifying patterns of shared meaning, review and refinement of the themes in relation to the coded extracts and the entire dataset, and finally definition and naming of themes. An overview of the analytical process is presented in Table 1. To support integration with the quantitative findings, the rich qualitative material was condensed into one overarching theme, “Between trust and uncertainty: family caregivers navigating a new model of care”, and two main themes, “Adapting to a new care context” and “Thriving, wellbeing, and diversity in caregiver experiences”, capturing key aspects of family caregivers’ experiences.

**Table 1:** Overview of the analytical process.

Overarching theme	Main theme	Code	Illustrative quotations
Between trust and uncertainty: family caregivers navigating a new model of care	Adapting to a new care context	First impressions and expectations	“We experienced great relief when the place became available.”
			“This is not an ordinary nursing home.”
		Transition experiences	“It was a dignified and warm welcome.”
			“I can finally sleep at night.”
			“They do a very good job. Many of them are incredibly kind.”
		Trust, uncertainty and communication	“The permanent staff are warm and welcoming.”
			*“*We feel that we are taken seriously and receive good follow-up*.”*
			“We asked them to follow things up, but it simply wasn’t followed through.”
		Changing caregiver roles	“I wanted to be seen as a partner, not only as a relative.”
			“It is still our role to make sure that things are functioning properly.”
			“I had to contact the physiotherapist myself.”
		Collaboration and involvement	“I feel included.”
			“The staff are approachable and understanding.”
			“I think we should have been much more involved.”
		Freedom, safety and technology	“The freedom is important.”
			“It was wonderful that she could move around without getting lost.”
			“The alarm failed.”
	Thriving, wellbeing, and diversity in caregiver experiences	Resident wellbeing and quality of life	“She has a sense of calm and security.”
			“I can see that she feels safe.”
			“He sits alone at a table.”
		Environment and meaningful activities	“Now there is life and activity.”
			“We always take two or three walks on the rooftop terrace.”
			“All the facilities are there, but they are closed.”
		Resources	“They try as best they can.”
			“Most of the staff are very attentive.”
		Coping	“It has been an enormous support.”
			“We have become closer through this.”
			“There is a lot of loss. There is grief.”
		Individual differences	“Activities should match the person and the life they have lived.”
			“She has always been physically active.”
			“He can become overlooked because his needs differ from those of other residents.”

**Table 2:** Characteristics and health status of family caregivers (*N* = 30).

Variable	Category	*n*	%	Mean	SD
Age (years)	30–39	1	3		
	40–49	2	7		
	50–59	15	50		
	60–69	2	7		
	≥70	10	33		
Sex	Female	19	63		
	Male	11	37		
Education	High school	4	13		
	Higher education (<4 years)	9	30		
	Higher education (≥4 years)	15	50		
Employment stats	Full-time	18	60		
	Part-time	2	7		
	Retired	9	30		
	Other	1	3		
Income, NOK	<599,000	8			
	600,000–999,999	12			
	<1,000,000	7	23		
Living locally	Yes	24	80		
	No	6	20		
Relationship to Resident	Spouse/partner	11	37		
	Son/daughter	16	53		
	Sibling	2	7		
	Other	1	3		
Visiting	Every day	1	3		
	2–3 times a week	11	36		
	Once a week	13	43		
	Twice a month	4	13		
	Once a month	1	3		
EQ-5D	Mobility	29		4.97	0.2
	Self-care	29		5	0
	Usual activities	29		4.66	0.7
	Pain/discomfort	29		4.34	0.7
	Anxiety/depression	29		4.48	0.7
	EQ VAS (0–100)	30		81.1	14

Values are presented as *n* (%) unless otherwise stated. For EQ-5D-5L dimensions, scores range from 5 (no problems) to 1 (extreme problems), with higher scores indicating better health status. EQ VAS scores range from 0 (worst imaginable health) to 100 (best imaginable health).

### Reflexivity

According to Braun and Clarke (2022), reflexivity is important in qualitative studies and should be maintained throughout the research process to enhance the study’s credibility and transparency. The researchers continuously reflected on how their professional backgrounds, prior experiences in dementia care and research, and preconceptions could influence data collection, interpretation, and theme development. Regular discussions within the research team encouraged critical reflection, challenged emerging interpretations, and supported the development of themes that remained grounded in participants’ accounts while acknowledging the researchers’ active role in the analytic process.

Meleis’ Transition Theory (Meleis, 2010) and the intersectional perspective (Hengelaar et al., 2023) were introduced later in the research process, after the initial inductive analysis and theme development were complete. Consequently, neither framework guided data collection, coding, or the inductive development of themes. Instead, they were applied as complementary interpretive lenses during the later stages of analysis and discussion. Meleis’ Transition Theory supported a deeper understanding of participants’ experiences of transition, while the intersectional perspective helped illuminate how intersecting social positions, identities, relationships, and contextual conditions could shape these experiences differently. The researchers remained reflexively attentive to the possibility that applying these perspectives could privilege particular interpretations. They were therefore used to illuminate and contextualise the findings rather than to reshape the themes or impose predetermined theoretical meanings on participants’ accounts.

### Ethical considerations

The study was approved by the Regional Committees for Medical and Health Research Ethics (REK 806176). Given the potential power imbalance inherent in recruiting family caregivers of residents living in DVs, the researchers sought to minimise any perceived pressure to participate by emphasising that participation was voluntary, their shared experiences were confidential, and independent of the care provided to their family member. Participants were also informed of their right to withdraw from the study at any stage without consequences for the care the resident would receive. The researchers were attentive to some participants’ advanced ages and health status throughout the interviews. When and if needed, breaks were offered to provide an opportunity to rest and ensure that participants remained comfortable and able to share their experiences.

All participants received written and oral information about the study and provided written informed consent prior to participation. Data were anonymised and stored securely in accordance with applicable ethical and data protection regulations from the researchers’ institutions. To protect participants’ anonymity, we did not report detailed demographic information for the qualitative sample. Given the relatively small number of family caregivers associated with each DV, presenting specific characteristics such as age, sex, or relationship to the resident could increase the risk of identification. Furthermore, although the sample included both family members and a small number of other informal caregivers, these characteristics are not linked to individual quotations or findings to maintain confidentiality.

## Results

### Participant and resident characteristics

Participants were aged 18 years or older and had a relative residing in one of the participating DVs. A total of 30 family caregivers participated in the study. The sample primarily comprised women, adult children, and spouses/partners of residents. Most participants had completed higher education, were employed, and visited the resident at least weekly. Self-reported health was generally good.

Residents were predominantly older adults, and most had lived with dementia for several years before moving to the DV. The majority had transitioned directly from their own homes. At the time of data collection, most residents had lived in the DV for 3–12 months, while about one quarter had resided there for more than 1 year (Tables 1–3).

**Table 3:** Characteristics of the residents.

Variable	Category	*n*	%
Age (years)	<60	2	7
	60–69	3	10
	70–79	10	33
	80–89	12	40
	>90	3	10
Time since diagnoses	<1 year	4	13
	1–2 years	3	10
	2–3 years	7	23
	>3 years	14	46
	unknown	2	7
How long in DV	<3 months	4	13
	3–9 months	11	37
	10–12 months	8	27
	>12 months	7	23
Transferred from	Home	21	70
	Other institution	9	30

Family caregivers reported high levels of caregiver esteem and generally low levels of burden related to family support, finances, and health, as presented in Table 4.

**Table 4:** Caregiver reaction assessment (CRA) (*N* = 30).

CRA dimension	Number of items	Possible range	Mean	SD
Caregiver esteem	7	1–5	3.9	0.4
Lack of family support	5	1–5	2.2	0.8
Impact on finances	3	1–5	2.1	0.9
Impact on schedule	5	1–5	2.9	0.9
Impact on health	4	1–5	2.3	0.6

All items are rated on a 5-point Likert scale ranging from 1 (*strongly disagree*) to 5 (*strongly agree*). Higher scores indicate a greater presence of the construct being measured. Selected items are reverse-scored according to the CRA scoring manual (Items 3, 7, 13, 15, and 19).

Overall, family caregivers perceived residents as thriving in the DV. Positive ratings were observed across all TOPAS-Proxy domains. Ratings were highest for staff relationships, whereas meaningful activities received the lowest scores among the domains assessed, as presented in Table 5.

**Table 5:** Thriving of older people assessment scale (TOPAS-Proxy) (*N* = 30).

Domain	Number of Items	Scale	Mean	SD
Attitude toward place of living	4	1–6	4.0	1.18
Quality of care and support	3	1–6	4.1	1.1
Staff relationships	8	1–6	4.7	0.9
Relationships with co-residents	3	1–6	4.1	1.1
Meaningful activities	6	1–6	3.9	1.2
Contact with people and places outside the facility	3	1–6	4.1	1.1
Physical environment	5	1–6	4.1	1.1
TOPAS total score	32	1–6	4.3	0.9
Overall thriving rating	1 item	1–10	6.89	2.5

Higher scores indicate higher levels of thriving. The overall thriving item is scored separately on a 10-point scale ranging from 1 (*strongly disagree*) to 10 (*strongly agree*).

### Correlations between overall thriving and TOPAS-proxy and CRA domains

Overall thriving was positively correlated with all TOPAS-Proxy domains. The strongest correlations were observed for relationships with co-residents (rho = 0.852, *p* < 0.001), quality of care and support (rho = 0.832, *p* < 0.001), and attitudes toward the place of living (rho = 0.812, *p* < 0.001). Significant positive correlations were also found for the physical environment (rho = 0.736, *p* < 0.001), meaningful activities (rho = 0.719, *p* < 0.001), staff relationships (rho = 0.655, *p* < 0.001), and contact with people and places outside the facility (rho = 0.474, *p* = 0.017). No significant correlations were found between overall thriving and any of the CRA domains (*p* > 0.05). Within the CRA, significant positive correlations were identified between impact on finances and impact on health (rho = 0.705, *p* < 0.001), and between impact on schedule and impact on health (rho = 0.666, *p* < 0.001), suggesting that family caregivers experiencing greater financial strain and disruption of daily activities also reported greater health-related burden.

### Qualitative findings

The qualitative findings are based on 16 semi-structured interviews with family caregivers from both DVs. Although the sample included family members as well as a small number of non-family caregivers, the term family caregivers is used throughout for consistency.

### Between trust and uncertainty—family caregivers navigating a new model of care

The overarching theme captures family caregivers’ experiences of adapting to the transition from providing care at home to supporting a relative living in a DV. Entering this new model of care involved an ongoing process of negotiating trust, redefining caregiving roles, and adjusting expectations of both the care environment and their continued involvement. While many family caregivers described relief and reassurance as responsibility for daily care was transferred to staff, they also experienced uncertainty regarding communication, decision-making, and balancing residents’ autonomy with safety. Their accounts illustrate that adapting to DV care was not a single event, but a dynamic process shaped by evolving relationships with residents, staff, and the care environment.

#### Adapting to a new care context

The transition from home, or previous care settings, to the DV was frequently described as emotionally demanding and characterised by uncertainty. For some family caregivers, particularly those who had experienced prolonged stress related to cognitive decline, behavioral challenges, or insufficient support at home, admission to the DV represented a major relief and provided a renewed sense of security.

“My mother won the lottery when she got a place here. It is physically designed to support activity and independence.” (Daughter)

“We experienced enormous relief when the place was offered.” (Son)

Although most family caregivers ultimately expressed trust in the care provided and viewed the DV as a supportive and dignified environment, several described a mismatch between expectations of the concept and their actual experiences. Some mentioned that the model had been presented in an overly positive manner, while others reported insufficient information before and during admission, contributing to uncertainty and frustration.

Family caregivers generally expressed satisfaction with the quality of care, which is consistent with the relatively high ratings for care and support and staff relationships in the quantitative findings. Nevertheless, many remained actively involved in their relative’s daily life and continued to experience emotional and practical burdens. Caregiving responsibilities did not disappear following DV residency but were transformed into new forms of involvement.

“The only thing I felt was relief at no longer being responsible throughout the night.” (Wife)

“They do a very good job. Many of them are incredibly kind.” (Daughter)

“It is still our role to make sure that things are functioning properly.” (Son)

Many participants expressed a desire for greater inclusion in care planning and decision-making and described partnership with staff as important for maintaining trust and continuity of care.

“I would very much like to be regarded as a partner [in care], not simply a relative.” (Daughter)

“I would have liked to know more, yes. But who was supposed to tell me, I do not know.” (Husband)

While several family caregivers reported positive, responsive collaboration, others described challenges related to communication and follow-up. Some said staff did not always act on or consistently follow information about residents’ needs and preferences. Participants also highlighted unclear admission and onboarding procedures, particularly during the early phase after the DVs opened, which contributed to uncertainty regarding roles, expectations, and communication pathways.

Balancing resident autonomy and safety emerged as a central concern. Family caregivers generally valued welfare technologies and the DV design for supporting both independence and security. However, trust in technology was sometimes challenged.

“It was wonderful because she could walk around freely without getting lost.” (Husband)

“The alarm failed, and they found him the next morning with a fractured hip.” (Wife)

“There is a monitoring camera in there as well … and that gives me a sense of security.” (Brother)

A few family caregivers described experiences in which falls were not detected by camera-based night-time monitoring, while others reported technical failures or difficulties using the technology. In contrast, some family caregivers expressed that the available technology was insufficient and purchased additional devices themselves to enhance safety and communication. Concerns regarding staffing levels, particularly during evenings and weekends, further influenced perceptions of safety and quality of care.

##### Thriving, wellbeing, and diversity in caregiver experiences

The physical and social environment of the DV was generally described as a major strength. Homelike surroundings, access to outdoor spaces, opportunities for meaningful activities, and the possibility of personalising rooms were perceived as promoting residents’ wellbeing, dignity, quality of life, and thriving. These accounts align with the positive ratings across TOPAS domains and the strong associations between overall thriving and perceptions of care, social relationships, and the physical environment.

“The first thing I thought was that he would have the opportunity to walk. He has always enjoyed walking.” (Brother)

“Now there is life and activity around her when she leaves her room.” (Daughter)

Several family caregivers particularly valued opportunities for residents to move freely within the DV, especially for those who had been physically active before admission. However, experiences varied by residents’ previous living arrangements and family caregivers’ resources and support networks. While family caregivers of residents who had moved from their own homes often viewed the DV as providing increased opportunities for social engagement and activity, some family caregivers of residents transferred from other nursing homes missed aspects of the previous setting, particularly its location and accessibility.

Organised activities, cultural events, volunteer involvement, and dedicated spaces supporting residents’ interests were frequently highlighted as important contributors to wellbeing and thriving.

“There is a lot happening there.” (Son)

“There are bonfires, choir activities, and many different events taking place.” (Wife)

At the same time, some family caregivers mentioned that activities were too limited or insufficiently tailored to personal preferences, particularly for male residents. Others described periods when communal facilities and amenities operated with reduced opening hours, leaving parts of the DV quiet and lacking activity outside scheduled events. These observations align with the quantitative findings, in which meaningful activities received lower ratings than other thriving domains.

“None of those expectations were fulfilled. There were no activities.” (Son)

“I think he could use much more activity — not just five minutes on the exercise bike.” (Son)

Participants also identified challenges related to the DV’s design, including complex layouts, limited signage, and variation in residents’ support needs. Some described a need for more clearly defined or protected areas, while others raised concerns about how the composition of resident groups influenced everyday life. Limited support from other family members contributed to feelings of isolation and increased caregiving burden for some participants. Despite these challenges, family caregivers consistently stated that they would choose the DV model again for their relatives, describing residents as safe, engaged, thriving, and well cared for.

“Compared with the alternatives that exist, I think this is the best option.” (Son)

## Discussion

This study explored family caregivers’ experiences with DV care and examined associations between caregiver reactions, perceived resident thriving, and key aspects of the DV environment. Overall, participants reported positive perceptions of resident thriving, high levels of caregiver esteem, and relatively low levels of caregiver burden. The qualitative findings complemented these results by highlighting both positive experiences with the DV model and ongoing challenges related to transitions, communication, involvement, and the balance between residents’ autonomy and safety.

The quantitative findings demonstrated significant correlations between overall thriving and all TOPAS domains, with the strongest correlations observed for relationships with co-residents, quality of care and support, and attitudes towards the living environment. These findings support previous TOPAS research describing thriving as a multidimensional phenomenon that reflects how individuals adapt to and experience wellbeing within their living environment (Bergland et al., 2014; Santana-Berlanga et al., 2024). Previous studies have highlighted the importance of social relationships, quality of care, environmental characteristics, and engagement in meaningful activities for promoting thriving and wellbeing among nursing home residents (Bergland et al., 2015; Björk et al., 2017; Santana-Berlanga et al., 2024). The present qualitative findings consistently describe family caregivers highlighting supportive staff, social connectedness, homelike surroundings, and opportunities for meaningful engagement as important contributors to residents’ wellbeing.

The present findings also contribute to a broader understanding of thriving within dementia care. Whereas quality of life is often conceptualised as an individual outcome, thriving appears to capture the dynamic interaction between residents, family caregivers, staff, and the care environment. The mixed-methods findings suggest that environmental design or care quality alone cannot explain thriving. Rather, it emerges through the interplay of meaningful activities, supportive relationships, opportunities for autonomy, and family caregivers’ confidence that residents are living meaningful everyday lives. This broader understanding may explain why family caregivers perceived residents as thriving even when they themselves continued to experience elements of burden.

Beyond describing positive relationships, the findings suggest that trust may function as a central mechanism through which the DV model supports both resident thriving and caregiver confidence. Rather than depending solely on individual staff members, trust appeared to emerge from the interaction between continuity of care, the small-scale organisation of the village, opportunities for informal communication, and a shared person-centred philosophy (Ekman et al., 2011). These organisational characteristics may help explain why many family caregivers expressed reassured despite continuing to experience responsibility for their relatives. At the same time, when communication or involvement was perceived as insufficient, this trust became more fragile, illustrating that thriving depends not only on the physical environment but also on the quality of ongoing relationships between families and healthcare professionals. Similar findings have been reported in previous dementia care research, in which positive relationships with staff did not necessarily prevent concerns about communication and family involvement (Richardson et al., 2019).

Research on dementia-friendly communities further suggests that supportive relationships are most effective when combined with organisational structures that promote inclusion, participation, and access to information (Craig et al., 2024). The importance of organisational factors is further supported by recent evidence from the French DV Village Landais Alzheimer Henri Emmanuelli (Krier et al., 2026). Compared with residents in traditional nursing homes, DV residents experienced fewer and shorter hospitalisations and were less likely to die in hospital. Moreover, these outcomes were related to features embedded in the DV model, including higher staffing levels, stronger interdisciplinary healthcare presence, continuity of care, and a homelike, person-centred environment.

The present findings are consistent with previous research from Norwegian DVs (Lillekroken et al., 2026), in which healthcare personnel identified resident autonomy, access to outdoor spaces, and meaningful activities as key features of the care model, while also highlighting the challenges of balancing residents’ autonomy with safety. Similar concerns were expressed by family caregivers, particularly regarding supervision, staffing, and care technologies, suggesting that supporting autonomy requires continuous negotiation in everyday practice. In this context, advanced monitoring technologies (e.g., GPS, electronic access bracelet, sensor-based cameras) are highly valued for facilitating resident autonomy and promoting freedom of movement within the DV. However, our findings regarding occasional undetected falls or system limitations suggest that the practical success of this “digital safety net” depends on its operational integration. Rather than replacing physical presence, welfare technology is most effective when conceptualized as an auxiliary, relational tool that enhances and supports human care (Gjerstad et al., 2025). Ensuring that digital monitoring is systematically paired with adequate physical staffing, particularly during overnight and weekend shifts, is essential for maintaining both resident safety and the relational foundation of person-centred dementia care. Our findings may indicate that thriving in DVs depends less on the presence of organised activities and more on whether activities are experienced as meaningful, personalised, and socially engaging. Together, they indicate that both relational and organisational dimensions of care are important for supporting positive family caregiver experiences and resident wellbeing.

Meaningful activities received lower ratings than other thriving domains and emerged as a recurring theme in the interviews. While the DV model is philosophically and structurally designed to integrate naturalistic, everyday activities (Roberts, 2023), our findings reveal a discrepancy in how consistently or visibly family caregivers experienced these practices. Although caregivers appreciated organised activities, cultural events, and outdoor opportunities, some felt these were not always sufficiently tailored to residents’ preferences, while others described the DV as quiet outside structured events. To enhance the visibility and clinical relevance of these activities, family caregivers highlighted three key strategies. First, facilities should establish formal structures to co-design activities with families and systematically leverage their biographical knowledge. Second, although the model intends for residents to actively participate in everyday domestic tasks like meal preparation or baking, family caregivers noted that those with advanced cognitive decline often remained unintegrated, highlighting the need for structured, adaptive facilitation to match individual capacities. Finally, activities should align more closely with residents’ prior professional and gender-specific identities, particularly by introducing structured physical or technical routines for male residents to prevent social withdrawal. These findings also indicate that meaningful engagement remains an area for further development, despite generally positive evaluations of the DV model overall. This is consistent with previous research on dementia-friendly and small-scale homelike care models, which identifies social participation and meaningful everyday activities as key contributors to quality of life and wellbeing, while highlighting the need for further research on outcomes for residents and their families (Krier et al., 2023).

An interesting finding was the absence of significant correlations between overall thriving and caregiver burden. Although family caregivers reported varying levels of burden, these experiences did not appear to influence their assessments of residents’ thriving. At the same time, financial burden, schedule burden, and health burden were strongly associated with one another, indicating that different dimensions of caregiver burden tend to cluster. Together, these findings suggest that family caregivers may distinguish between their own experiences of caregiving and their perceptions of residents’ wellbeing. Similarly, results from a study conducted by Quinn et al. (2019) indicate that both caregiving stress and positive caregiving experiences were independently associated with caregiver wellbeing and life satisfaction when examined in the same model. The authors argued that caregiving should be understood as a multidimensional experience in which positive and negative appraisals may coexist rather than representing opposite ends of a single continuum. Consistent with this perspective, family caregivers in the present study may have experienced substantial burden while nevertheless perceiving that their relatives were thriving in the DV environment.

Viewed through Meleis’ Transition Theory (Meleis, 2010), these findings suggest that the move to a DV represents more than a physical relocation. It also involves a gradual transition in family caregivers’ identities, responsibilities, and relationships with healthcare professionals. Successful transitions appeared to depend not only on the quality of care itself but also on opportunities for information sharing, dialogue, and role negotiation. These findings therefore extend previous applications of Transition Theory by illustrating how adaptation continues well beyond admission and is shaped through everyday interactions within the DV environment (Khalifeh et al., 2026). Together, the findings suggest that the transition to DV care is an ongoing process that requires attention to both practical and emotional support needs. This ongoing adaptation may also explain why family caregivers frequently described increasing confidence over time despite initial uncertainty. Rather than representing a fixed outcome, thriving appeared to develop through successful transitions characterised by trust, collaboration, and continuity of care.

An intersectional perspective (Hengelaar et al., 2023) suggests that family caregivers’ experiences were shaped not by single characteristics such as age or relationship to the resident, but by the interaction among multiple social and contextual positions. The findings suggest considerable variation in how family caregivers experienced the transition to DV care. Burden related to daily schedules was somewhat higher than the other burden dimensions, indicating a busy daily life. Thus, differences in caregiving circumstances, available support, and individual situations appeared to shape how family caregivers managed trust, involvement, uncertainty, and evolving caregiving roles within the DV context.

As populations age and the number of people living with dementia increases, we argue that the DV care model is important because it represents more than an alternative architectural design or residential setting; it reflects a reorientation of dementia care toward continuity of everyday life, social inclusion, autonomy, and personhood. Although relocation to a DV still involves leaving one’s previous home, the community-like environment may enable residents to remain socially embedded by supporting familiar routines, movement, relationships, and participation in meaningful everyday activities. This is consistent with research showing that naturalistic social environments can strengthen older adults’ sense of belonging, social identity, and quality of life (Achmad and Zaluchu, 2024). Our findings further suggest that this model’s value extends beyond residents. When family caregivers perceive their relatives as safe, socially connected, and able to live a recognisable everyday life, they may experience greater reassurance and reduced emotional strain. DV may therefore contribute simultaneously to residents’ well-being and family caregivers’ emotional sustainability. In contemporary dementia care, their significance lies in challenging highly controlled, institutional approaches and showing how long-term care can provide necessary support and safety without unnecessarily restricting social participation, agency, and meaningful living. Nevertheless, further comparative and longitudinal research is required to determine how consistently these potential benefits are achieved across different settings and populations.

Finally, the findings suggest that DVs may contribute to more sustainable dementia care. Although family caregivers continued to provide emotional support, practical assistance, and advocacy following admission, most described the DV positively and stated that they would choose the model again. Similar findings have been reported in previous DV research, where relatives remained actively involved in creating and maintaining meaningful everyday lives for residents despite the availability of formal care (Peoples et al., 2020; Krier et al., 2026). Sustainability in DV care may therefore depend not only on the physical environment and the principles of person-centred care, but also on the ability of the model to facilitate constructive partnerships between formal and informal caregivers. When family caregivers are informed, involved, and supported throughout the care trajectory, DVs may promote sustainable outcomes for both residents and families over time.

### Implications for clinical practice

The findings suggest that DVs as a model of care should continue to promote meaningful collaboration with family caregivers after admission, recognising their ongoing knowledge of and emotional connection with the resident. Healthcare professionals should facilitate regular communication, involve family caregivers in care planning and decision-making when appropriate, and acknowledge their evolving caregiving role. Supporting trusting partnerships between staff and family caregivers may enhance residents’ wellbeing and thriving while contributing to more person-centred and sustainable dementia care.

The residents’ perceived navigational challenges highlight a broader, fundamental design tension inherent to the DV model: balancing a homelike, non-institutional environment with clear environmental cues for people with progressive cognitive impairment. In traditional nursing homes, clinical, highly visible signage and uniform lighting often support environmental navigation but compromise the domestic aesthetic. DVs intentionally reject these clinical signposts to foster a natural, homelike environment. However, our findings indicate that without carefully integrated visual and spatial landmarks, de-institutionalisation can lead to disorientation and loss of autonomy. To resolve this design tension, future DV developments should merge domestic aesthetics with subtle, evidence-based cognitive and physical environmental cues, ensuring that environmental legibility is maintained without introducing a clinical or institutional feel.

### Strengths and limitations

This study has several strengths and limitations. One strength is the mixed-methods design. The use of TOPAS-Proxy, a theory-based instrument developed and psychometrically evaluated to assess older people’s thriving in relation to their living environment. The instrument has demonstrated satisfactory validity and reliability for proxy-rated use in nursing home settings and enabled a systematic assessment of multiple dimensions of thriving (Baxter et al., 2019; Bergland et al., 2014, 2015). Furthermore, integrating the TOPAS-Proxy results with qualitative interviews provided a more comprehensive understanding of residents’ thriving from the perspective of family caregivers. Nevertheless, proxy ratings should be interpreted as relational assessments that may reflect not only caregivers’ observations of residents but also their own experiences of burden, relief, anxiety, or satisfaction following the transition to dementia village care.

By triangulating data sources, first collecting quantitative data and subsequently conducting qualitative interviews, it was possible to further explore, explain, and contextualise the results from the survey, providing a more comprehensive understanding of family caregivers’ experiences of DV care. The qualitative sample included family caregivers from two DVs with varied relationships to residents, contributing diverse perspectives while maintaining a shared experience of the DV care model. The sample was considered to provide sufficient information power to address the study aim, given its specificity and the richness of participants’ accounts (Malterud et al., 2016).

Another strength of the study was the use of Meleis’ Transition Theory and an intersectionality perspective as complementary theoretical lenses for interpreting and discussing the findings. Meleis’ Transition Theory helped us understand the move to DV care as an ongoing process involving changes in family caregivers’ roles, responsibilities, relationships, and identities, rather than a single event ending their caregiving role. The intersectionality perspective further sensitised us to how caregivers’ experiences may be shaped by the interaction of multiple social and contextual factors, such as gender, relationship to the resident, caregiving history, available resources, and expectations surrounding family responsibility. Together, these perspectives supported a more nuanced interpretation of the findings by illuminating both the transitional processes caregivers experienced and the circumstances contributing to variation in their experiences. However, we recognise that researchers applying other theoretical perspectives may interpret and emphasise different aspects of the findings. The interpretations presented should therefore be understood as theoretically situated rather than as the only possible explanation of family caregivers’ experiences.

Several limitations should also be considered when interpreting the findings. The relatively small quantitative sample (*n* = 30) limits statistical power and generalisability. Additionally, resident thriving was assessed through caregiver proxy reports, which may be subject to a positive appraisal bias. Following institutionalisation, family caregivers often experience substantial physical and emotional relief from the intensive demands of home-based care (e.g., describing admission as a “major relief” allowing them to “finally sleep”). This newfound relief may unconsciously incline family caregivers to rate the residents’ thriving more favourably. While the strong convergence between our quantitative proxy ratings and qualitative interview data supports the credibility of the findings, future studies should triangulate proxy reports with direct observational measures to control for potential emotional and role-transition biases. Furthermore, the qualitative findings are based on data from family caregivers in two Norwegian DVs and should therefore be interpreted in light of the specific organizational and cultural context. Although the findings may resonate with similar care settings, readers should assess their transferability to other contexts.

Member checking was not undertaken. In reflexive thematic analysis, knowledge is co-constructed between participants and researchers, and themes reflect interpretative understandings rather than objective accounts. Consequently, returning themes to participants for verification was not considered, as it is inconsistent with the epistemological assumptions underpinning this analytic approach (Braun and Clarke, 2022).

## Conclusion

The transition into DV care appears to be an ongoing process rather than a single event. While family caregivers generally perceived residents as thriving and expressed confidence in the care provided, the findings highlight the importance of communication, involvement, and support throughout the transition. Social relationships, quality of care, meaningful engagement, and the care environment emerged as key factors associated with thriving. Strengthening collaboration between family caregivers and staff may further support positive experiences for both residents and their families and contribute to the long-term sustainability of DV care.

## Data Availability

The raw data supporting the conclusions of this article will be made available by the authors, without undue reservation.
